# Massive Upper Gastrointestinal Bleeding From a Duodenal Diverticulum in a Nonagenarian: A Report of a Rare Case

**DOI:** 10.7759/cureus.95340

**Published:** 2025-10-24

**Authors:** Rohan Tariq, Aiman Rashid, Muhammad Adil Zaka Khan

**Affiliations:** 1 Gastroenterology and Hepatology, East Kent Hospitals University NHS Foundation Trust, Ashford, GBR; 2 Gastroenterology and Hepatology, Queen Elizabeth The Queen Mother Hospital, Margate, GBR

**Keywords:** adrenaline therapy, diverticular bleeding, duodenal ulceration, git endoscopy, hemoclips, upper gastrointestinal (ugi) bleeding

## Abstract

Duodenal diverticula are typically asymptomatic and incidentally discovered, but they can rarely lead to significant complications, including upper gastrointestinal (UGI) bleeding. We report the case of a 91-year-old woman who presented with melaena and haematemesis. Endoscopy revealed an actively bleeding duodenal diverticulum, which was successfully managed with endoscopic haemostasis. This case illustrates the importance of considering duodenal diverticula as a rare cause of UGI bleeding in elderly patients and highlights the role of prompt endoscopic diagnosis and treatment.

## Introduction

Duodenal diverticula are typically asymptomatic and incidentally discovered, but they can rarely lead to significant complications, including upper gastrointestinal (UGI) bleeding [1]. While relatively common, especially in older populations, they rarely present with bleeding. When they do, the bleeding can be severe and potentially life-threatening, particularly in the elderly. We report the case of a 91-year-old woman who presented with melaena and haematemesis. Endoscopy revealed an actively bleeding duodenal diverticulum, which was successfully managed with endoscopic haemostasis [2]. This case illustrates the importance of considering duodenal diverticula as a rare cause of UGI bleeding [3] in elderly patients and highlights the role of prompt endoscopic diagnosis and treatment.

Duodenal diverticula are out-pouchings of the duodenal wall and are most often asymptomatic [4]. Diagnosis can be challenging due to the diverticula's location in the distal duodenum, which is often overlooked during routine upper endoscopy.

## Case presentation

A 91-year-old woman presented to the emergency department with a two-day history of black tarry stools and one episode of haematemesis. She reported generalised fatigue but denied abdominal pain, fever, or recent NSAID (nonsteroidal anti-inflammatory drug) use. Her past medical history included hypertension and osteoarthritis. On examination, she was pale, hypotensive (blood pressure 90/60 mmHg), and tachycardic (heart rate 110 bpm). Abdominal examination was unremarkable. Digital rectal examination confirmed melaena.

Investigations

Initial blood tests showed a low haemoglobin level of 6 g/dL (baseline: 11 g/dL), a normal platelet count, and a normal coagulation profile. Blood urea nitrogen was elevated at 10.5 mmol/L (normal upper limit 7.8 mmol/L), consistent with an upper GI source of bleeding. A Glasgow Blatchford Score of 14 indicated a high risk of bleed [4].

**Figure 1:**
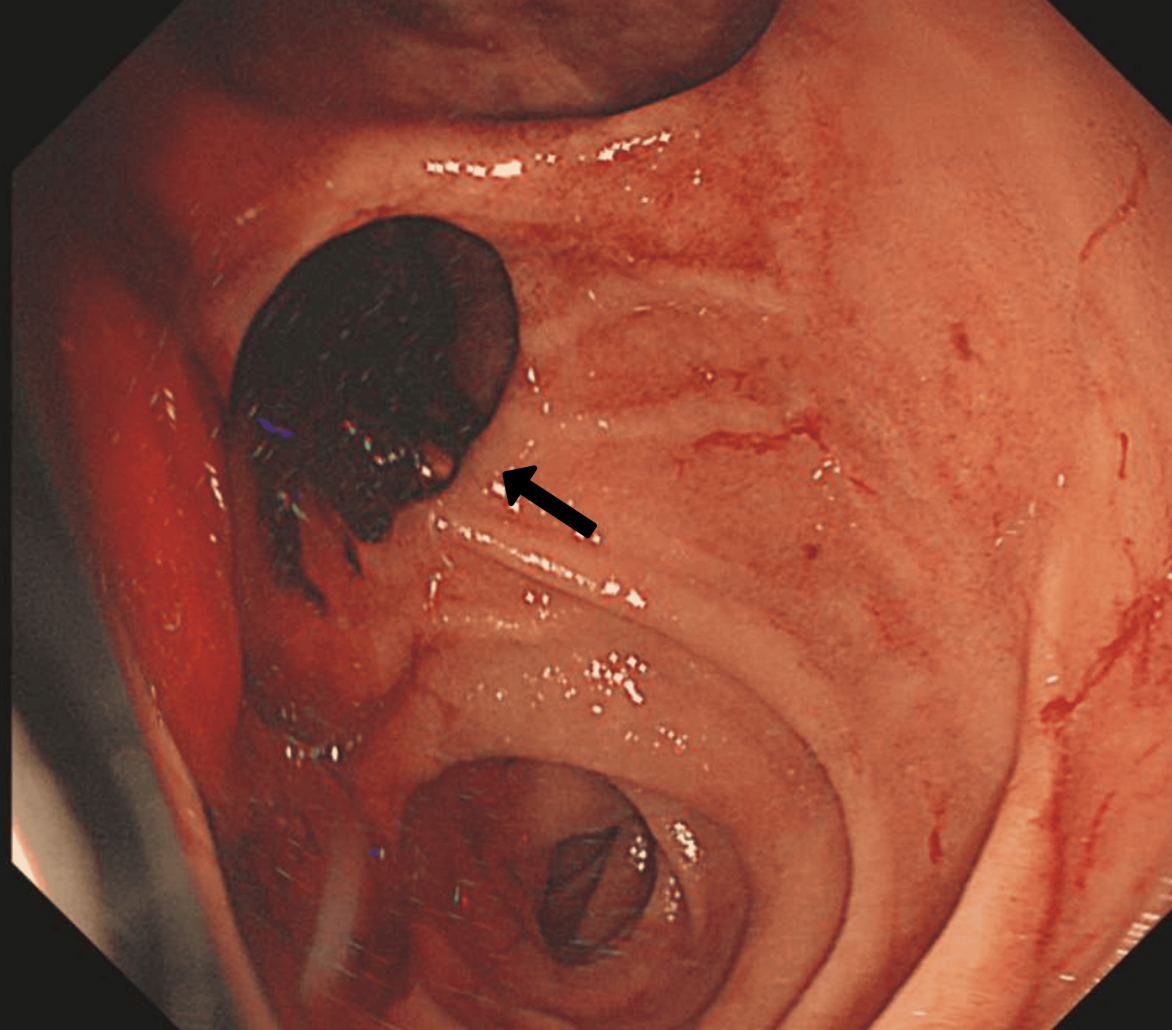
View of the second part of the duodenum showing a duodenal diverticulum with pooling of blood (as indicated by the black arrow) during the oesophagogastroduodenoscopy

**Figure 2:**
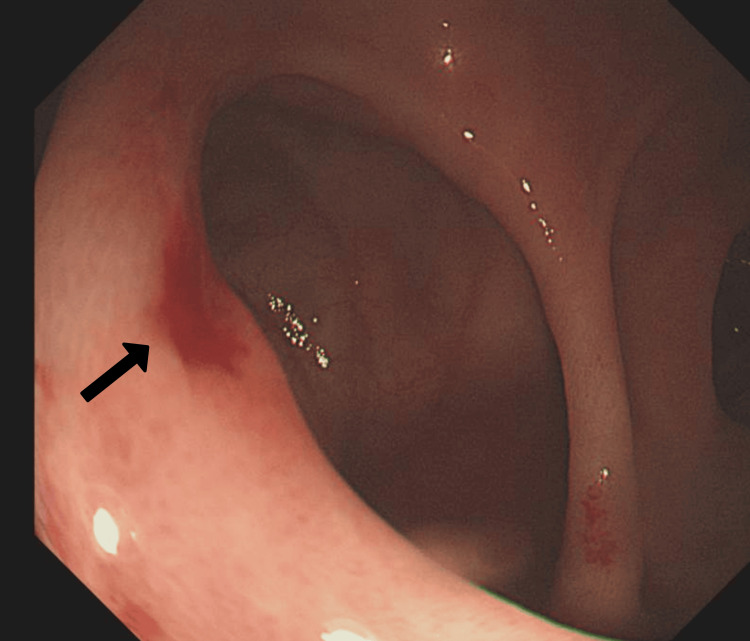
Forrest IIc ulcer – flat pigmented haematin on ulcer base (pointed by the black arrow)

After resuscitation with intravenous fluids and packed red blood cells, an urgent oesophagogastroduodenoscopy (OGD) was performed. The OGD revealed a large duodenal diverticulum in the second part of the duodenum (Figure 1) with a Forrest IIc ulcer [5] at the junction (Figure 2). No varices or gastric lesions were observed.

Treatment

**Figure 3:**
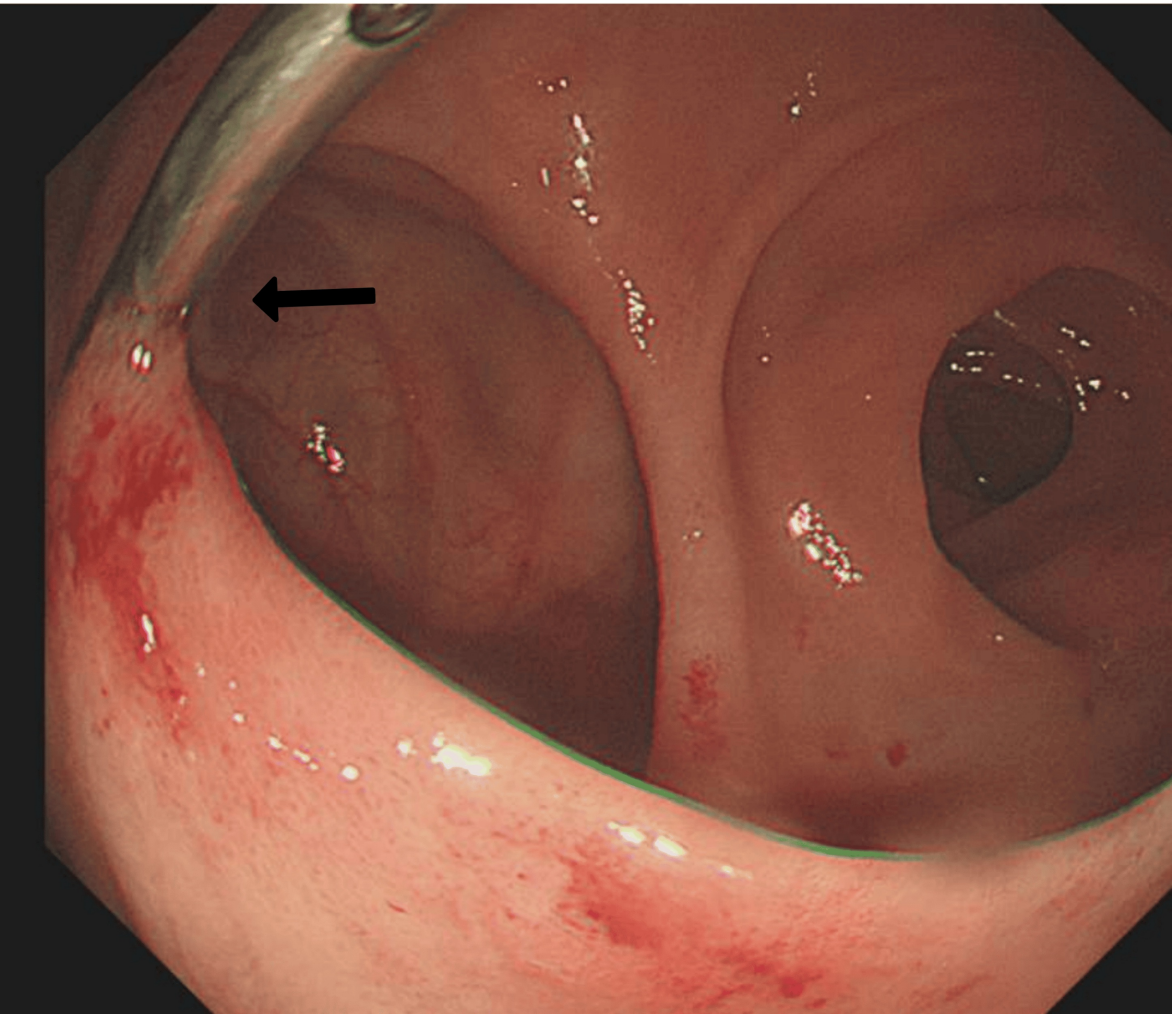
Insertion of an endoclip at the ulcer site (pointed by the arrow) to achieve haemostasis

Endoscopic haemostasis was achieved using 6 ml of adrenalin 1/10000 via five injections, followed by the placement of one haemoclip (Figure 3) [6].

The patient was monitored in the high-dependency unit post-procedure. She was kept nil-by-mouth initially and gradually resumed oral intake after 48 hours. No further bleeding episodes occurred during hospitalisation [7].

Outcome and follow-up

The patient remained haemodynamically stable and showed good recovery. Repeat haemoglobin levels remained stable, and there was no recurrence of bleeding. She was discharged on day six with outpatient gastroenterology follow-up and advice to avoid NSAIDs.

## Discussion

Although duodenal diverticula are found in up to 22% of the population [8], complications such as bleeding are rare (<5%) [9]. When bleeding occurs, it is often due to erosion of a vessel at the base of the diverticulum. In elderly patients, multiple co-morbidities and atypical presentations can delay diagnosis.

Endoscopy is the diagnostic modality of choice, but diverticula may be missed if the second or third portion of the duodenum is not adequately visualised. In cases of active bleeding, endoscopic therapy using injection, thermal coagulation, or clipping is usually successful. Surgical or radiological intervention is reserved for refractory cases.

This case demonstrates the importance of thorough endoscopic evaluation and maintaining a high index of suspicion for rare causes of bleeding in elderly patients [10].

## Conclusions

Duodenal diverticula, though usually asymptomatic, can rarely cause significant upper GI bleeding. Thorough examination of the distal duodenum during OGD is crucial, particularly when initial findings are negative. Endoscopic therapy is effective in achieving haemostasis in bleeding duodenal diverticula. Consider duodenal diverticula as a differential in elderly patients with obscure or recurrent upper GI bleeding.
